# Development and Evaluation of a Paper-Based Microfluidic Device for Detection of *Listeria monocytogenes* on Food Contact and Non-Food Contact Surfaces

**DOI:** 10.3390/foods11070947

**Published:** 2022-03-25

**Authors:** Codi Jo Broten, John B. Wydallis, Thomas H. Reilly, Bledar Bisha

**Affiliations:** 1 Department of Animal Science, University of Wyoming, Laramie, WY 82071, USA; cbroten@uwyo.edu; 2 Access Sensor Technologies, Fort Collins, CO 80524, USA; johnwydallis@gmail.com (J.B.W.); tomreilly@accsensors.com (T.H.R.III)

**Keywords:** diagnostics, colorimetric detection, foodborne pathogens, PCR, rapid detection, environmental sampling

## Abstract

*Listeria monocytogenes* is the third most deadly foodborne pathogen in the United States. The bacterium is found in soil and water, contaminating raw food products and the processing environment, where it can persist for an extended period. Currently, testing of food contact and non-food contact surfaces is performed using an array of sampling devices and endpoint technologies, offering various levels of sensitivity, cost, user skill, and time to detection. Paper-based microfluidic devices (µPADs) are a rapid detection platform amenable to low-cost, user-friendly, and portable diagnostics. In this study, we developed and evaluated a µPAD platform specific for the colorimetric detection of the *Listeria* genus following recovery from food contact and non-food contact surfaces. For detection, four colorimetric substrates specific for the detection of β-glucosidase, two broths selective for the detection of *Listeria* spp., and a nonselective broth were evaluated to facilitate detection of *Listeria* spp. The limit of detection and time to detection were determined by using pure bacterial cultures. After 8 h enrichment, *L. monocytogenes* (10^2^ Colony Forming Units (CFU)/coupon) was detected on every surface. After 18 h enrichment, *L. monocytogenes* (10^2^ CFU/coupon) was detected on all surfaces with all swabbing devices. This study demonstrated the ability of the µPAD-based method to detect potentially stressed cells at low levels of environmental contamination.

## 1. Introduction

The Centers for Disease Control and Prevention (CDC) estimates that foodborne illness afflicts one in six Americans annually, equating to 48 million cases, 128,000 hospitalizations, and 3000 deaths [1]. *Listeria* spp. are Gram-positive rods, facultative anaerobic, non-spore-forming, and non-encapsulated [2]. *Listeria monocytogenes* is the etiological agent of an estimated 1600 incidences of foodborne illness and a resultant 260 deaths annually in the United States [3]. Of concern to the food safety industry is the ability of *L. monocytogenes* to grow at refrigeration temperature, as well as the ability to tolerate acidic and high-salt conditions [4,5].

One listeriosis infection is associated with approximately $1.7 million in medical expenses [6]. Listeriosis is frequently linked to consumption of adulterated soft cheeses, deli meats, hot dogs, raw sprouts, melons, unpasteurized milk, and smoked fish [7]. *L. monocytogenes* is ubiquitously distributed throughout the environment and is often found in soil and water [5]. 

A zero-tolerance policy for *L. monocytogenes* in ready-to-eat (RTE) meat and poultry products is enforced by the United States Department of Agriculture Food Safety and Inspection Service (USDA FSIS), driving the need for effective detection efforts [4]. Detection of *Listeria* species is used as an indicator for the presence of *L. monocytogenes* in food and the environment [8]. Prompt detection of *Listeria* spp. is crucial, as the bacterium can persist for an estimated 10 years in a processing facility [4]. A sampling regimen is necessary, as *L. monocytogenes* can persist in harborage sites, such as drains [9].

Regulatory sampling regimens make use of a multitude of sampling devices and protocols for environmental and food contact surface sampling. The Food and Drug Administration’s Bacteriological Analytical Manual (FDA BAM) method requires a 24–48 h enrichment or the use of alternate screening methodologies. If employed, the alternate screening methodology must be approved for environmental surface sampling. Alternate screening methodologies for detection of *Listeria* spp. in environmental surface samples include immunoassays requiring prior enrichment [10]. The USDA FSIS *Listeria* guide approves the use of assays targeting *L. monocytogenes*, *Listeria* spp., or *Listeria*-like organisms for detection on food contact and non-food contact surfaces. An enrichment step is typically performed to recover injured cells and increase target analytes to a detectable level, followed by a screening method validated by a regulatory agency or scientific association (ex: FDA BAM, AOAC, etc.) [9].

Detection of *Listeria* spp. may be performed rather than the detection of *L. monocytogenes* because there is no USDA FSIS requirement for confirmation of *L. monocytogenes* after detection of *Listeria* spp.; however, corrective action must be taken. Non-food contact surfaces may be included in a sampling plan, although they are not required, to indicate inadequate sanitation [9].

Paper-based diagnostics are an attractive option as a rapid detection platform. These platforms can be paired with culture-based [11], immunological [12], and molecular detection of analytes [13] for rapid testing. Conveniently, µPADs can serve as a screening device for the detection of pathogens in the food industry [14].

Several studies have found differences in detection and recovery of environmental *L. monocytogenes* depending on surface(s), sampling devices, and level of inoculum used, including Lahou & Uyttendale and Vorst et al. [15,16]. However, previous studies detecting low levels of environmental *L. monocytogenes* are limited [15]. Even more importantly, studies coupling environmental sampling with µPAD-based detection are scarce.

β-glucosidase activity, also referred to as esculin hydrolysis, depending on the substrate employed, is often used to differentiate *Listeria* spp. from other organisms, such as *Escherichia coli* and other Enterobacteriaceae including *Salmonella*. β-glucosidase is also produced by *Enterococcus* spp., *Serratia* spp. [17], and *Bacillus* spp. [18], thus necessitating the use of selective enrichment methods for detection of *Listeria* spp. Colorimetric detection is an attractive format commonly used by chromogenic agars and can be paired with paper-based microfluidic devices (µPADs) for enzymatic detection.

The objective of this study was to develop and validate a rapid *Listeria* genus specific test to detect this bacterium on food contact and non-food contact surfaces using a µPAD platform. To this end, the µPAD platform was paired with culture-based enrichment and colorimetric detection.

## 2. Materials and Methods

### 2.1. Manufacture of µPADs

The µPAD was designed using Adobe Illustrator CS6 (version 19.2.1, San Jose, CA, USA) and printed on Whatman No. 4 paper (Marlborough, MA, USA) using a Xerox ColorQube 8570 (Norwalk, CT, USA). Wax was melted through the paper using a Puhui T-962C infrared oven (Tai’an, China) at 140 °C for 5 min. The sheet of µPADs was laminated using the Akiles Prolam Photo 6 Roller Laminator (Loma, CA, USA) with a 9 × 11.5^2^ laminating film pouch (3 mil (75 mic), Pfeiffer, Crows Nest, Australia). The laminate was cut around the µPAD devices using a Trotec Speedy 100 CO_2_ laser (Plymouth, MI, USA).

### 2.2. Bacterial Culture

*Listeria monocytogenes* Scott A, *Listeria innocua* ATCC 51742, *Bacillus pumilus* ATCC 700814, and *Escherichia coli* ATCC 25922 strains were maintained at −80 °C in Brain Heart Infusion broth (HiMedia, Mumbai, India) with 20% (vol/vol) glycerol (Amresco, Radnor, PA, USA). Cultures were activated by streaking onto Brain Heart Infusion agar (HiMedia, Mumbai, India) and incubated aerobically at 37 °C overnight. Individual colonies were picked, and triplicate portions of 10 mL Brain Heart Infusion broth were inoculated, then aerobically cultured at 37 °C and allowed to reach a pre-determined OD_600_.

A 1.0 mL portion of culture was centrifuged at 5000× *g* for 5 min at room temperature (Sorvall Legend Micro 17, Thermo Scientific, Waltham, MA, USA), washed once with Phosphate Buffered Saline pH 7.2 (PBS, BD Diagnostics, Franklin Lakes, NJ, USA), and resuspended in an equal volume.

For the preparation of bacterial cultures for surface sampling, washed cultures of *L. monocytogenes* and *B. pumilus* were diluted to prepare cultures of *L. monocytogenes* containing 10^1^, 10^2^, and 10^3^ Colony Forming Units (CFU)/mL, and *B. pumilus* containing 10^3^ CFU/mL.

### 2.3. Substrate Concentration Evaluation

Four substrates, indoxyl-β-d-glucoside (plant indican, ACROS Organics, Waltham, MA, USA), 5-bromo-4-chloro-3-indolyl-β-D-glucopyranoside (x-glc, Alfa Aesar, Ward Hill, MA, USA), 5-bromo-6-chloro-3-indolyl-β-D-glucopyranoside (magenta-glc, CHEM-IMPEX INT’L INC, Wood Dale, IL, USA), and 6-chloro-3-indolyl-β-d-glucopyranoside (salmon-glc, Biosynth Chemistry & Biology, Staad, Switzerland), were evaluated for the detection of β-glucosidase, an enzyme indicative of *Listeria* spp. Stock solutions of x-glc, magenta-glc, and salmon-glc were produced using the solvent dimethyl sulfoxide (DMSO, Fisher Chemical, Waltham, MA, USA) and manufacturer reference solubility guidelines (see Table S1). A stock solution of plant indican was produced using Milli-Q water (Millipore Sigma, Burlington, MA, USA) as the solvent and manufacturer reference solubility guideline (see Table S1). All stock solutions were produced in triplicate to account for chemical replication, and four two-fold dilutions were produced in identical solvent to yield five concentrations of each substrate. Stock solutions were stored in dark conditions at 4 °C.

Substrate evaluation was performed on µPADs. The cultures *L. monocytogenes*, *L. innocua*, and *B. pumilus* were maintained and incubated as detailed in the bacterial culture step above, except that they were cultured for 18 h. The experiment was repeated in biological triplicate. The optimal substrate concentration was calculated as the largest average greyscale intensity for *L. monocytogenes* using ImageJ analysis.

### 2.4. Incubation of µPAD and Sample

The µPADs, placed in 100 × 15 mm Petri dishes, were impregnated with 1 µL of substrate, allowed to dry in a laminar flow hood at room temperature, then spotted with 30 µL of sample. Petri dishes containing µPADs impregnated with substrate and sample were wrapped with Parafilm M (Neenah, WI, USA) and incubated aerobically at 37 °C until dry.

### 2.5. ImageJ Analysis

Dry µPADs were scanned using a HP Deskjet 3510 scanner (Palo Alto, CA, USA) and saved as 300 dpi TIFF images. Images were analyzed using ImageJ software. In ImageJ, the following settings were utilized: image → type → 32-bit and edit → invert. The oval icon tool was used to select the individual µPAD well. The measure setting under the analyze tab was used to calculate greyscale intensity and was manually recorded from ImageJ.

### 2.6. Evaluation of Microbiological Media Optimal for μPAD Detection

Two selective broths for *Listeria* spp., Listeria Enrichment Media (Actero FoodChek System Inc., Calgary, AB, Canada) and ONE Broth-Listeria (Oxoid, Basingstoke, England), and a non-selective enrichment medium, Brain Heart Infusion broth, were evaluated for use. As both *Listeria* spp. and *Bacillus* spp. exhibit β-glucosidase activity, a selective broth is required to enhance selectivity. All broths (10 mL portions) were inoculated to contain 1 CFU/mL and 100 CFU/mL of *L. monocytogenes*, *L. innocua*, *B. pumilus*, and *E. coli*, and an overnight culture of *L. monocytogenes*. The evaluation of microbiological media was performed in biological triplicate. Cultures were grown aerobically at 37 °C for 18 h. Aliquots were added to a µPAD impregnated with x-glc. The threshold for further testing was determined using the results from ImageJ analysis. The average greyscale intensity plus 2× standard deviation of 1 CFU/mL and 100 CFU/mL of *B. pumilus* and *E. coli* cultures grown in Listeria Enrichment Media was calculated as the threshold value.

### 2.7. Determination of Limit of Detection (LOD) and Time to Detection (TTD)

Triplicate portions of 10 mL Listeria Enrichment Media were inoculated to contain 1, 10, 10^2^, and 10^3^ CFU/mL of *L. monocytogenes*, then incubated aerobically at 37 °C for 18 h. Aliquots removed at time 0, 4, 8, and 18 h were added to a µPAD impregnated with x-glc and analyzed by ImageJ when dry.

### 2.8. Preparation of Sampling Surfaces

Stainless-steel (grade 304), neoprene rubber (commercial grade 65A), and high-density polyethylene (HDPE, type 300) was obtained and cut into 10 × 10 cm coupons. The cleaning protocol was adapted and modified from [19]. For HDPE and rubber, coupons were sprayed with 70% ethanol and allowed to dry at room temperature in a laminar flow hood for 1 h. For stainless-steel, coupons were wrapped with tinfoil and taped closed before autoclaving at 121 °C for 25 min. After an experiment, all coupons were sprayed with 70% ethanol, washed with Liquinox (Alconox, White Plains, NY, USA), and rinsed with deionized water.

The inoculation procedure was adapted and modified from [16]. Three of each coupon type were spotted with 100 µL of the respective bacterial inocula, which were spread using a sterile disposable cell spreader (Fisherbrand, Waltham, MA, USA). Inoculated coupons were allowed to dry in a laminar flow hood at room temperature for 1 h before surface sampling. The experiment was repeated with triplicate bacterial cultures.

### 2.9. Calcium Alginate Swab, Rayon-Tipped Swab, and Sponge-Stick Sampling Methods

Sterile individually wrapped calcium alginate swabs (Puritan, Guilford, ME, USA) and rayon-tipped swabs (Puritan, Guilford, ME, USA), both with wooden handles, were wetted in 10 mL of Listeria Enrichment Media and gently wiped along the inside of the tube. Swabbing patterns were modeled after [15]. Coupons were sampled with calcium alginate swabs and rayon-tipped swabs using a rolling “S” pattern to cover the entire coupon surface in the horizontal, vertical, and diagonal directions. The tip of the swab was used to sample the perimeter of the coupon. The microbial detachment procedure was adapted from [16]. After surface sampling, the device was placed back into the tube of Listeria Enrichment Media, the handle was broken off, and tubes were vortexed at 3000 rpm (Fisherbrand, Waltham, MA, USA) for 1 min.

Sterile individually wrapped dry sponge-sticks (3M, Maplewood, MN, USA) were wetted in 10 mL Listeria Enrichment Media and wringed of excess liquid. Swabbing patterns were modeled after [15]. The device was moved in a “S” pattern to cover the entire coupon surface in the horizontal direction and flipped to the opposite side to cover the vertical and diagonal directions. The edge of the sponge was used to sample the perimeter of the coupon. The microbial detachment procedure was adapted and modified from [16]. After surface sampling, the device was placed back in the included bag, an additional 10 mL Listeria Enrichment Media was added, and the device was processed by maceration (Seward Tekmar lab blender 400, Norfolk, UK) for 1 min.

### 2.10. Surface Sampling Enrichment

All swabbing devices were incubated aerobically at 37 °C, with aliquots removed at 0, 8, and 18 h. Aliquots were kept refrigerated until samples from all time points were collected each day. Aliquots were spotted in duplicate (*n* = 648) onto a µPAD impregnated with x-glc and quantitated using ImageJ. Aliquots were stored at −80 °C until DNA extractions were performed.

### 2.11. PCR-Based Confirmation

PCR was used for confirmation. The iQ-Check *Listeria monocytogenes* II PCR Detection Kit (Bio-Rad, Hercules, CA, USA) was used for DNA extraction with modifications to enrichment protocol. The samples from the 18 h enrichment timepoint which were positive using ImageJ analysis and the *B. pumilus* negative control samples from the 18 h enrichment timepoint were used for the DNA extraction protocol. The standard protocol for DNA extraction was used, with the exception that vortexing at 3000 rpm for 3 min replaced processing by cell disruptor. Extracted DNA was stored at −20 °C.

Real-time PCR was performed on a CFX 96 (Bio-Rad, Hercules, CA, USA) using the CFX Manager Software (version 3.1., Bio-Rad, Hercules, CA, USA). The *Listeria monocytogenes* II PCR Detection Kit amplification settings were utilized for real-time PCR. After an initial 10 min step at 95 °C to activate polymerase, a 15 s denaturation step at 95 °C, 20 s annealing step at 58 °C, and 30 s extension step at 72 °C were repeated for a total of 50 cycles.

### 2.12. Statistics

The GLIMMIX procedure of SAS (version 9.4, SAS Inst. Inc., Cary, NC, USA) was used to determine mean differences for the evaluation of substrates, broths, and TTD and LOD. The pdiff function was employed to evaluate all pairwise differences. The GLIMMIX procedure of SAS was utilized to evaluate the effect of surface, swab, and treatment mean differences at time 0, 8, and 18 h enrichment on greyscale intensity. The pdiff function was used to evaluate all pairwise differences. The GENMOD procedure of SAS was utilized to evaluate the PCR method compared to the µPAD method for correct detection of positive and negative controls. The sensitivity, specificity, positive predictive value (ppv), and negative predictive value (npv) were calculated according to [20]. An α of 0.05 was considered as significant for all statistical procedures.

## 3. Results

### 3.1. Substrate Concentration Evaluation

**Figure 1:**
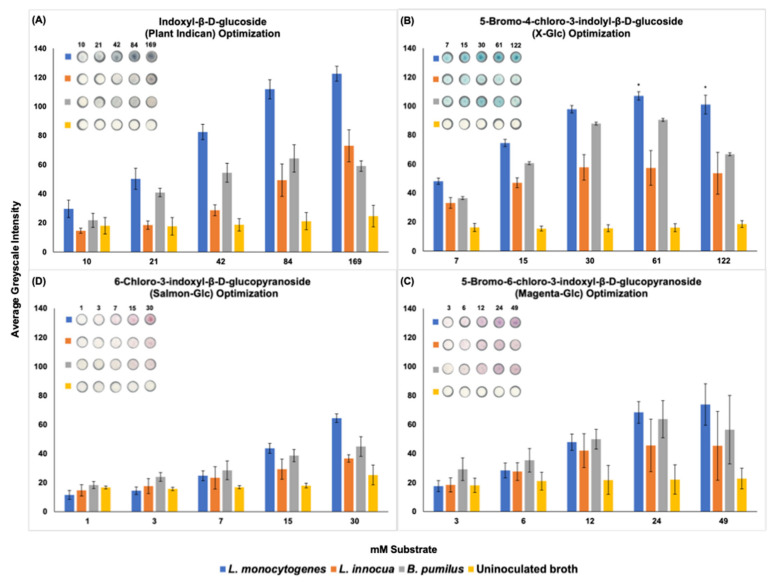
Evaluation of substrates specific for detection of β-glucosidase activity. The average greyscale intensity was determined using ImageJ. Bars show standard deviations of three means. Asterisks (*) represent statistical significance for evaluation of *L. monocytogenes* and x-glc (*p* < 0.0001). Digitized colorimetric images. Representative wells are depicted. (**A**) plant indican, (**B**) x-glc, (**C**) magenta-glc, (**D**) salmon-glc. From left to right on the x-axis is smallest to largest substrate concentration tested in mM.

A µPAD platform paired with four colorimetric substrates for β-glucosidase activity revealed that plant indican produced the largest average greyscale intensity (122.55) for *L. monocytogenes*. In the evaluation of substrate, the plant indican solution rapidly discolored to a deep indigo color. To mitigate the discoloration, the solution was wrapped in tinfoil and stored at both 4° and −20 °C. Unfortunately, these strategies were not sufficient to prevent rapid discoloration, thus necessitating the selection of a different substrate for detection of β-glucosidase activity. The evaluation of x-glc with *L. monocytogenes* produced the second largest average greyscale intensity and was thus chosen for inclusion as the substrate for the remaining development phases. The highest concentration of x-glc, 122 mM, was chosen for the remaining steps of the experiment to ensure the adequate distribution of the substrate. The greyscale intensity means of 122 mM x-glc (101.10) and 62 mM x-glc (107.09) were statistically the same. The average greyscale intensity produced by x-glc and *L. monocytogenes* was 1.36× and 1.57× greater than the average greyscale intensity produced by magenta-glc and salmon-glc, respectively (Figure 1).

### 3.2. Evaluation of Microbiological Media Optimal for µPAD Detection

**Figure 2:**
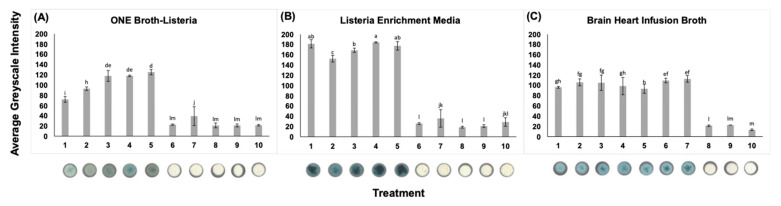
Evaluation of microbiological media for the development of a test specific for the detection of *Listeria* spp. Target *L. monocytogenes* was detected using every broth tested. Non-target *E. coli* and *B. pumilus*, the latter of which produces the target enzyme β-glucosidase, were both inhibited by the selective enrichment broths ONE Broth-Listeria and Listeria Enrichment Media. The average greyscale intensity was determined using ImageJ. Bars show standard deviations of three means. Means with different letters are significantly different. Digitized colorimetric images. Representative wells are depicted. (**A**) ONE Broth-Listeria, (**B**) Listeria Enrichment Media, (**C**) Brain Heart Infusion broth. From left to right on the x-axis are the following treatments: (1) *L. monocytogenes* 1 CFU/mL, (2) *L. monocytogenes* 100 CFU/mL, (3) *L. monocytogenes* overnight culture, (4) *L. innocua* 1 CFU/mL, (5) *L. innocua* 100 CFU/mL, (6) *B. pumilus* 1 CFU/mL, (7) *B. pumilus* 100 CFU/mL, (8) *E. coli* 1 CFU/mL, (9) *E. coli* 100 CFU/mL, (10) uninoculated broth.

Broths selective for *Listeria* spp. were employed side by side with a nonselective broth using positive and negative controls for β-glucosidase production, as well as an uninoculated broth control. Nonselective Brain Heart Infusion broth allowed for the growth of all organisms tested, as expected. Selective Listeria Enrichment Media and ONE Broth-Listeria suppressed growth and subsequent detection of *B. pumilus* and *E. coli*, while allowing growth of *Listeria* spp., as per the medium specifications. The average greyscale intensity of Listeria Enrichment Media was significantly greater than all other broths evaluated for *L. monocytogenes*, and was therefore employed for all further testing. The average greyscale intensity produced by 1 CFU/mL *L. monocytogenes* grown in Listeria Enrichment Media was 1.88× and 2.52× greater than the average greyscale intensity produced by Brain Heart Infusion broth and ONE Broth-Listeria, respectively (Figure 2). The threshold for greyscale intensity was calculated as 35.6.

### 3.3. Determination of LOD and TTD

**Figure 3:**
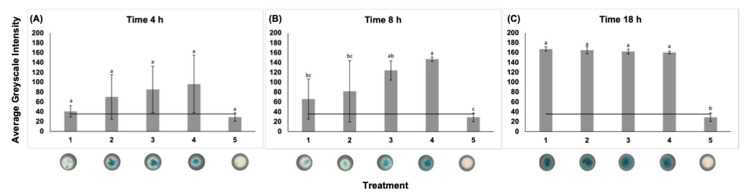
LOD and TTD for the development of a test specific for the detection of *Listeria* spp. The average greyscale intensity was determined using ImageJ. The threshold value is represented in black. Bars show standard deviations of three means. Means with different letters are significantly different. Digitized colorimetric images. Representative wells are depicted. (**A**) time 4 h, (**B**) time 8 h, (**C**) time 18 h. From left to right on the x-axis are the following treatments: (1) *L. monocytogenes* 1 CFU/mL, (2) *L. monocytogenes* 10 CFU/mL, (3) *L. monocytogenes* 10^2^ CFU/mL, (4) *L. monocytogenes* 10^3^ CFU/mL, (5) uninoculated broth.

On average, all concentrations of *L. monocytogenes* tested were detected after 4 h enrichment. The average greyscale intensity of 1 CFU/mL increased 2.38× and 6.02× after eight and 18 h enrichment, respectively (Figure 3).

### 3.4. L. monocytogenes Surface Sampling

After 8 h enrichment, *L. monocytogenes* 10^2^ CFU/coupon was detected on all surfaces, albeit not with every swabbing device employed. The µPAD results were clearly discernable upon visual inspection, as well as following ImageJ analysis. After 8 h enrichment, *L. monocytogenes* was detected on 22% (6/27) of surface, swab, and treatment combinations. At this time point, *L. monocytogenes* 10^2^ CFU/coupon was detected on HDPE using a rayon-tipped swab and sponge-stick, on rubber using a calcium alginate swab, and on stainless-steel using all three swabbing devices.

**Figure 4:**
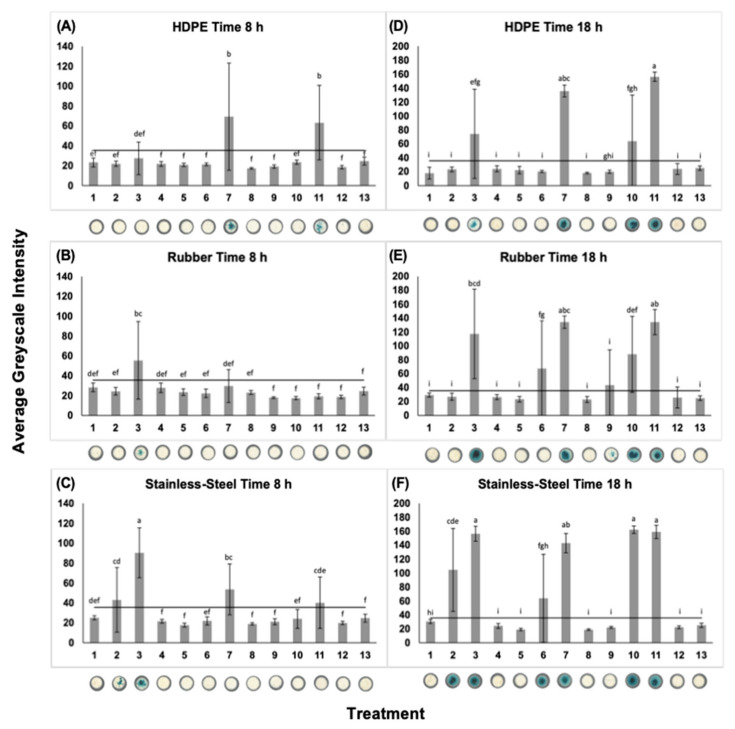
Detection of *L. monocytogenes* on food contact and non-food contact surfaces using enzymatic colorimetric detection paired with a µPAD platform. Enrichment time points at eight (left panel) and 18 h (right panel) are depicted. After 18 h enrichment, detection of *L. monocytogenes* on a µPAD platform is clearly discernable upon visual inspection. The average greyscale intensity was determined using ImageJ. The threshold value is represented in black. Bars show standard deviations of three means. Means with different letters are significantly different. Digitized colorimetric images. Representative wells are depicted. From top to bottom: (**A**,**D**) HDPE, (**B**,**E**) rubber, (**C**,**F**) stainless-steel. From left to right on the x-axis are the following treatments: (1) *L. monocytogenes* 1 CFU/coupon calcium alginate swab, (2) *L. monocytogenes* 10 CFU/coupon calcium alginate swab, (3) *L. monocytogenes* 10^2^ CFU/coupon calcium alginate swab, (4) *B. pumilus* 10^2^ CFU/coupon calcium alginate swab, (5) *L. monocytogenes* 1 CFU/coupon rayon-tipped swab, (6) *L. monocytogenes* 10 CFU/coupon rayon-tipped swab, (7) *L. monocytogenes* 10^2^ CFU/coupon rayon-tipped swab, (8) *B. pumilus* 10^2^ CFU/coupon rayon-tipped swab, (9) *L. monocytogenes* 1 CFU/coupon sponge-stick, (10) *L. monocytogenes* 10 CFU/coupon sponge-stick, (11) *L. monocytogenes* 10^2^ CFU/coupon sponge-stick, (12) *B. pumilus* 10^2^ CFU/coupon sponge-stick, (13) uninoculated broth.

After 18 h enrichment, *L. monocytogenes* 10^2^ CFU/coupon was detected on all surfaces with all swabbing devices and *L. monocytogenes* 10 CFU/coupon was detected on all surfaces with select swabbing devices. Following 18 h enrichment, *L. monocytogenes* was detected from 59% (16/27) of surface, swab, and treatment combinations. At the lowest concentration of *L. monocytogenes* tested (1 CFU/coupon), only cells from the stainless-steel with sponge-stick combination were detected. At the concentration of 10 CFU/coupon, *L. monocytogenes* was not detected on HDPE using a calcium alginate swab or rayon-tipped swab, or on rubber using a calcium alginate swab (Figure 4).

### 3.5. Simulated Food Surface, Swab, and Treament Effects

The effects of surface (*p* = 0.0346), swab (*p* = 0.0094), and treatment (*p* < 0.0001) were significant on greyscale intensity after 8 h enrichment. Specifically, the greyscale intensity produced by the stainless-steel effect was significantly greater than the rubber effect (*p* = 0.0097). The greyscale intensity detected after employment of the calcium alginate swab was significantly greater than from the rayon-tipped swab (*p* = 0.0468) and sponge-stick (*p* = 0.0027). For the treatment term, the greyscale intensity of *L. monocytogenes* 10^2^ CFU/coupon was significantly greater than all other treatments (*p* < 0.0001).

After 18 h enrichment, the effects of surface (*p* < 0.0001), swab (*p* < 0.0001), and treatment (*p* < 0.0001) were significant on greyscale intensity. Tests performed on stainless-steel had a 1.22× greater greyscale intensity than rubber (*p* = 0.0074) and a 1.52× greater greyscale intensity than HDPE (*p* < 0.0001). The tests performed using sponge-stick produced greyscale intensity that was 1.34× and 1.40× greater than the greyscale intensity from rayon-tipped swabs (*p* = 0.0001) and calcium alginate swabs (*p* < 0.0001), respectively. Specifically, after 18 h, enrichment of the greyscale intensity of tests performed on the *L. monocytogenes* 10^2^ CFU/coupon treatment was 5.92×, 5.27×, and 2× greater than the greyscale intensity of the test on *B. pumilus* 10^2^ CFU/coupon, *L. monocytogenes* 1 CFU/coupon, and *L. monocytogenes* 10 CFU/coupon treatment, respectively.

At the 8 h enrichment time point, the surface*swab (*p* = 0.0006) and surface*treatment (*p* = 0.0065) interaction effects were also significant. At the 18 h time point, the effect of swab*treatment (*p =* 0.0005) and surface*treatment (*p* < 0.0001) were significant on greyscale intensity detection. Of noteworthiness, the greyscale intensity of stainless-steel *L. monocytogenes* 10^2^ CFU/coupon was significantly greater than all other surface*treatment interactions (*p* < 0.0001).

### 3.6. PCR-Based Confirmation

For the detection of *L. monocytogenes*, the GENMOD procedure of SAS revealed no significant difference between detection using the µPAD platform and the PCR method (*p* = 0.2303). The GENMOD procedure of SAS revealed the µPAD and PCR methods were not significantly different for the detection of *B. pumilus* negative control (*p* = 0.0748). PCR of time 18 h *B. pumilus* samples and time 18 h positive µPAD samples revealed three false positive and zero false negative results. Calculated diagnostic statistics were as follows: 100% sensitivity, 89% specificity, 92% ppv, and 100% npv, respectively.

## 4. Discussion

In this study, we developed and evaluated a *Listeria* genus-specific test amenable to testing of food contact and non-food contact surfaces. Inocula were recovered using established surface sampling methods for facile and rapid detection, enriched, and detected using a µPAD platform coupled with colorimetric detection.

The detection of β-glucosidase activity using magenta-glc and salmon-glc produced a greyscale intensity many factors lower than x-glc, which was chosen for further testing due to a significantly greater greyscale intensity and easy discernment upon visual inspection.

Our results from this study are comparative to Klass et al. [21]. In their study, the Bio-Rad iQ Check *Listeria* spp. kit was modified for applied surface sampling using sponges with stainless-steel coupons and compared to a reference USDA method. Contamination levels of 68 CFU and 210 CFU *L. monocytogenes* were evaluated. Klass et al. reduced the volume of incubation for a sampling sponge, from 225 mL for the manufacturer’s protocol to 60 mL. We further reduced this volume to 20 mL for this study, without compromising results.

The fractional detection percentage (59% or 16/27) of *L. monocytogenes* after 18 h enrichment may be attributable to the adhesion of *Listeria* to the surface. Smoot & Pierson determined that *L. monocytogenes* Scott A in PBS at pH 7.0 adhered to rubber and stainless-steel at temperatures as low as 10 °C [22]. Adherence was quantified as early as after 10 min of exposure. Our LOD of 10^2^ CFU *L. monocytogenes* on all coupons with all swabbing devices was slightly lower than Limoges et al., who detected 99.1% of 1 CFU/cm^2^ (10^2^ CFU *L. monocytogenes*) and 100% of 1 CFU/cm^2^ on plastic and stainless-steel surfaces, respectively [23].

Our detection sensitivity is also attributable to the CFU per coupon. We inoculated coupons with 1, 10, and 100 cells per 100 cm^2^ coupon. Our study addresses the concern of sampling for and recovery of low levels of listerial environmental contamination. Other studies inoculated food contact surfaces with higher levels of listeriae, 10^2^ CFU/250 cm^2^ [15] and 10^5^ CFU/100 cm^2^ [24]. Nyachuba & Donnelly found 91.9% of a *Listeria* population is injured after exposure to 1 h drying time, identical to the protocol of our experiment [25]. Therefore, our recovery percentages should be considered in context to recovery of injured cells.

Lahou & Uyttendale observed a significant difference in the detection of 100 CFU/cm^2^ *L. monocytogenes* between stainless-steel, high-density polyethylene, and rubber surfaces when paired with cultural enrichment, followed by selective plating. However, there was no observed difference between sampling devices [15]. Vorst et al. employed four sampling devices for recovery of 10^6^ CFU/cm^2^ *L. monocytogenes* Scott A from stainless-steel. Recovered *L. monocytogenes* was detected via spiral plating with 48 h incubation. A composite tissue recovered significantly more *L. monocytogenes* than an environmental sponge, cotton-tipped swab, or calcium alginate swab. The environmental sponge recovered the least amount of *L. monocytogenes* [16].

For our detection of *L. monocytogenes* on food contact and non-food contact surfaces, the swab choice led to greater differences compared to surfaces sampled. Sponge-stick recovered inoculum that was detected using the µPAD platform at 10 and 10^2^ CFU/coupon *L. monocytogenes* on all surfaces tested. Sponge-stick also recovered 10 CFU/coupon *L. monocytogenes* on a rubber coupon. The significantly greater detection of *L. monocytogenes* using a sponge-stick aligns with the results of [26], who recovered the largest amount of viable *L. monocytogenes* using this sampling device. However, Vorst et al. discovered that *Listeria* may still be trapped in swabbing devices and a portion may remain on the contaminated surface [16]. Our imperfect limit of detection is likely attributable to stress and/or the effectiveness of the swabbing device.

As sampling and enrichment protocols from regulatory agencies vary for detection of *L. monocytogenes* from food contact and non-food contact surfaces, the protocol for this study incorporated elements from the USDA [27] and FDA BAM [10] reference methods. A merging of standard methods allows for the use of swabs for sampling in hard-to-reach areas and sponges for more accessible surfaces.

Established surface sampling methods for the detection of *L. monocytogenes* on food contact and non-food contact surfaces paired with colorimetric detection on a µPAD platform provided results in a shorter amount of time than the FDA BAM method for *Listeria* [10], as our method provided results after 18 h enrichment and the FDA method requires a 24–48 h enrichment period. Demonstration of µPAD platforms for detection of pathogens in the food industry is limited. Examples include [11,28,29,30], among others. This publication addresses gaps in the literature: the application of µPADs to a food processing environment and comparison to standard methods.

A µPAD device is constructed using chromatography paper with the creation of hydrophobic and hydrophilic zones. The inherent capillary action of paper drives fluid, removing the need for an external pump. Results are read using an external program or interpreted upon visual inspection [31]. Colorimetric detection methods paired with µPADs allow for visual qualitative confirmation (yes/no), as well as semiquantitative confirmation using an image capture tool, such as a scanner, digital camera, or mobile phone camera paired with imaging software [32].

The manufacture of µPADs is potentially amenable to the rapid production of suitable prototypes and mass production of devices for commercialization. A multitude of methods can be utilized to craft µPADs for diagnostic use, including photoresist [33], wax screen-printing [34], wax printing [35], three-dimensional crafting [36], wax pen, inkjet printing plus wax pen painting [37], and stamping [38]. Considerations in designing a µPAD assay for detection of foodborne pathogens should address the following challenges: pathogens are heterogeneously distributed at low levels, pathogens are often injured in a food or food processing environment, and detection of pathogens may be inhibited by background contribution [39].

The appeals of µPADs for diagnostics include the following: (1) µPADs are low-cost to manufacture, (2) multiplexing can be performed using µPADs, (3) implementation of µPADs requires less-skilled workers than other methods, and (4) colorimetric tests can be paired with cell phone remote analysis [40]. When µPAD diagnostics are employed with enzymatic detection, a catalytic colorimetric response is observed. Enzymatic detection paired with µPADs has been used to detect foodborne pathogens [29], to discriminate between pathogenic and nonpathogenic species [11], and to detect the presence of antibiotics [28].

Our sensitivity of 10^2^ CFU *L. monocytogenes* following 18 h enrichment is comparable to other microfluidic devices. Jokerst et al. detected 10^1^ CFU/mL *L. monocytogenes* on RTE meat after 12 h enrichment [29]. Chen et al. detected 1.6 × 10^2^ CFU/mL *L. monocytogenes* using a microfluidic biosensor paired with immunomagnetic separation. Lettuce was also inoculated with *L. monocytogenes* and 2.4 × 10^2^ CFU/mL was detected using a fluidic separation chip [41]. At present, we believe this is the first experiment that demonstrates proof of concept using a µPAD platform and detection of *L. monocytogenes* in representative surfaces of the processing environment. The µPAD device is portable, disposable, low-cost, and user-friendly.

The development of diagnostic tests is driven by the World Health Organization’s (WHO) ASSURED guidelines: Affordable, Sensitive, Specific, User-friendly, Rapid, Equipment-free, and Delivered to end users. The ASSURED diagnostic is ideally utilized in resource-limited settings. This paper serves as a model substrate for ASSURED diagnostic devices. While designing a µPAD device, the opportunities exist to create a test that is appropriate for off-site testing, lacks the need for continued maintenance, and that is scalable. Potentially, µPADs can be used for food safety and environmental remote testing to remove the need for the transport of samples. Sample preparation methods, including preconcentration of target and removal of matrix interference, and different reagent delivery approaches should be further explored in designing a µPAD prototype. Enhanced image capture, image remote analysis, and on device readout are areas likely to be explored in the future advancement of µPADs [40].

## Data Availability

The data presented in this study are available on request from the corresponding author.

## Supplementary Materials

The following are available online at https://www.mdpi.com/article/10.3390/foods11070947/s1, Table S1: Manufacturers of chromogenic substrates and reference solubility guidelines used to create stock solutions.
