# Supplementary material for: Development and Evaluation of a Paper-Based Microfluidic Device for Detection of Listeria monocytogenes on Food Contact and Non-Food Contact Surfaces
Source: Foods. 2022 Mar 25;11(7):947. doi: 10.3390/foods11070947 (PMC8997480; doi:10.3390/foods11070947)
Supplement: Supplementary file 1 [file foods-11-00947-s001.zip › foods-1633619-supplementary.pdf]

foods

Supplementary materials

Development and Evaluation of a Paper-Based Microfluidic Device for Detection of *Listeria monocytogenes* on Food Contact and Non-Food Contact Surfaces

Codi Jo Broten [1], John B. Wydallis [2], Thomas H. Reilly III [3] and Bledar Bisha [1,*]

[1] Department of Animal Science, University of Wyoming, Laramie, WY 82071, USA; cbroten@uwyo.edu
[2] Access Sensor Technologies, Fort Collins, CO 80524, USA; johnwydallis@gmail.com (J.B.W.); tomreilly@accsensors.com (T.H.R.III)
* Correspondence: bbisha@uwyo.edu

Table S1. Manufacturers of chromogenic substrates and reference solubility guidelines used to create stock solutions.

Name	Synonym	CAS #	MW (g/mol)	Maximum Solubility	Reference Solvent	Solvent Used	Solubility Source	Chemical Brand Used
Indoxyl-ß-D-glucoside	Plant indican	487-60-5	295.29	50 mg/mL	Water	MilliQ Water	Sigma	ACROS Organics
5-bromo-4-chloro-3-indolyl- ß-D-glucoside	X-glc	15548-60-4	408.63	50 mg/mL	DMF	DMSO	Sigma	Alfa Aesar
5-bromo-6-chloro-3-indoxyl-ß-D-glucopyranoside	Magenta-glc	93863-89-9	408.63	2% w/v	DMF	DMSO	Chemipex	CHEM-IMPEX INT'L INC.
6-chloro-3-indoxyl-ß-D-glucopyranoside	Salmon-glc	159954-28-6	329.73	1% w/v	Methanol	DMSO	saicarbohydrates.com	Biosynth Chemistry & Biology
